# Response to the Letter to the Editor by Dr Koratala et al

**DOI:** 10.1177/20543581251404700

**Published:** 2025-12-24

**Authors:** Guillaume Soret, Antonio Leidi, Thomas A. Mavrakanas

Re: Point-of-Care Ultrasonography and Worsening of Renal Function in Acute Heart Failure: A Cohort Study. Can J Kidney Health Dis. 2025 Jul 29; 12: 20543581251328069.

We would like to thank Dr Koratala et al for their interest in our manuscript and their thoughtful comments which highlight important aspects of our manuscript.^1^

Our main finding was the higher incidence of worsening renal function among patients with significant pulmonary congestion, as identified by an elevated number of B-lines on point-of-care ultrasound. We agree with their interpretation that worsening renal function in those individuals is most likely the consequence of elevated right cardiac filling pressures, resulting in venous congestion of the kidney. We also agree that a small rise in creatinine in patients with acute heart failure undergoing effective decongestion with appropriate diuretic therapy is benign, as shown in the DOSE trial and discussed in our manuscript. Furthermore, we agree that implementing a 28-point ultrasound protocol is not feasible in daily clinical practice, and our team has indeed shown that an 8-point protocol is feasible and reliable in assessing pulmonary congestion.^2^

The lack of association between worsening renal function and the tricuspid annular plane systolic excursion (TAPSE) or inferior vena cava (IVC) size on expiration should indeed be interpreted with caution, as it is most likely due to inadequate study power due to the small sample size. As Dr Koratala et al rightly point out, both TAPSE and IVC are imperfect measures. For example, patients in respiratory distress due to acute pulmonary edema may have significant collapsibility of their IVC, even though they are hypervolemic. It is true that Mitral Inflow Doppler and Doppler Tissue Imaging of Mitral Annular Motion are measurements that can be used to determine whether left ventricular filling pressures are elevated. In practice though, these measurements are time-consuming and require advanced ultrasound skills. Therefore, they might prove more challenging for physicians who have limited experience with point-of-care ultrasound.^3^ Perhaps 1 day, these measurements might be easier to perform with artificial intelligence-enabled tools, but studies to ensure the reproducibility and diagnostic accuracy of these techniques will still need to be carried out in the future.

Finally, we would like to provide some clarifications with respect to certain points raised by Dr Koratala et al. Although we agree that B-lines are not specific to acute pulmonary edema, this cohort only enrolled participants with a clinical diagnosis of acute heart failure and considerably elevated natriuretic peptides. Patients with other conditions which could affect the number of B-lines, such as interstitial lung disease or acute respiratory distress syndrome, were formally excluded from this study.^2^ Finally, ultrasonographers were not nephrologists but general internists with significant expertise in point-of-care ultrasound: all of them had conducted several hundreds of point-of-care scans prior to participating in this study and had received specific training in the study protocol for accurate counting of B-lines.


Guillaume Soret Division of General Internal Medicine, Department of Medicine, Geneva University Hospitals, Switzerland
Antonio Leidi Division of General Internal Medicine, Department of Medicine, Geneva University Hospitals, Switzerland
Thomas A. Mavrakanas Division of Nephrology, Department of Medicine, McGill University Health Center and Research Institute, Montreal, QC, Canada

